# Plasticity of the selectivity filter is essential for permeation in lysosomal TPC2 channels

**DOI:** 10.1073/pnas.2320153121

**Published:** 2024-07-29

**Authors:** Afroditi-Maria Zaki, Süleyman Selim Çınaroğlu, Taufiq Rahman, Sandip Patel, Philip C. Biggin

**Affiliations:** ^a^Department of Biochemistry, Structural Bioinformatics and Computational Biochemistry, University of Oxford, Oxford OX1 3QU, United Kingdom; ^b^Department of Pharmacology, University of Cambridge, Cambridge CB2 1PD, United Kingdom; ^c^Department of Cell and Developmental Biology, University College London, London WC1E, 6BT, United Kingdom

**Keywords:** conductance, sodium, lysosome, channel, twin-pore

## Abstract

Two-pore channels are pathophysiologically important Na^+^- and Ca^2+^-permeable channels expressed in lysosomes and other acidic organelles. Unlike most other ion channels, their permeability is malleable and ligand-tuned such that when gated by the signaling lipid PI(3,5)P_2_, they are more Na^+^-selective than when gated by the Ca^2+^ mobilizing messenger nicotinic acid adenine dinucleotide phosphate. However, the structural basis that underlies such plasticity and single-channel behavior more generally remains poorly understood. A recent Cryo-electron microscopy (cryo-EM) structure of TPC2 bound to PI(3,5)P_2_ in a proposed open-channel conformation provided an opportunity to address this via molecular dynamics (MD) simulation. To our surprise, simulations designed to compute conductance through this structure revealed almost no Na^+^ permeation events even at very high transmembrane voltages. However further MD simulations identified a spontaneous transition to a dramatically different conformation of the selectivity filter that involved expansion and a flip in the orientation of two core asparagine residues. This alternative filter conformation was remarkably stable and allowed Na^+^ to flow through the channel leading to a conductance estimate that was in very good agreement with direct single-channel measurements. Furthermore, this conformation was more permeable for Na^+^ over Ca^2+^. Our results have important ramifications not just for understanding the control of ion selectivity in TPC2 channels but also more broadly in terms of how ion channels discriminate ions.

## Significance

The textbook view of ion permeation is that ion channels have a fixed selectivity. Two Pore Channel 2 (TPC2) channels found on lysosomes are however an exception. Structural biology often provides insight into assigning a particular conformation to a corresponding electrophysiological state but this is not trivial. Here, we demonstrate that a previously suggested open-state structure is not permissive to ion flux, but that the filter can undergo an unexpectedly large conformational change that does allow sodium ions to permeate at a rate and permeability consistent with direct electrophysiological measurements. Our results demonstrate that selectivity filters in ion channel proteins may exhibit much more dynamic heterogeneity than first appreciated and suggest that there may be more than one way to discriminate ions in biology.

Lysosomes are central to recycling damaged cellular material, but it is clear now that they play numerous other functions including a critical role in ion homeostasis (1). TPCs are a family of ancient cation channels that localize to lysosomes and other acidic organelles where they regulate processes such as trafficking of endocytic cargo, membrane contact site formation, and osmotic balance (2–4). TPCs are implicated in liver dysfunction, neurodegeneration, and pigmentation disorders as well as other diseases and are therefore emerging as drug targets (2, 5–8).

**Fig. 1.**
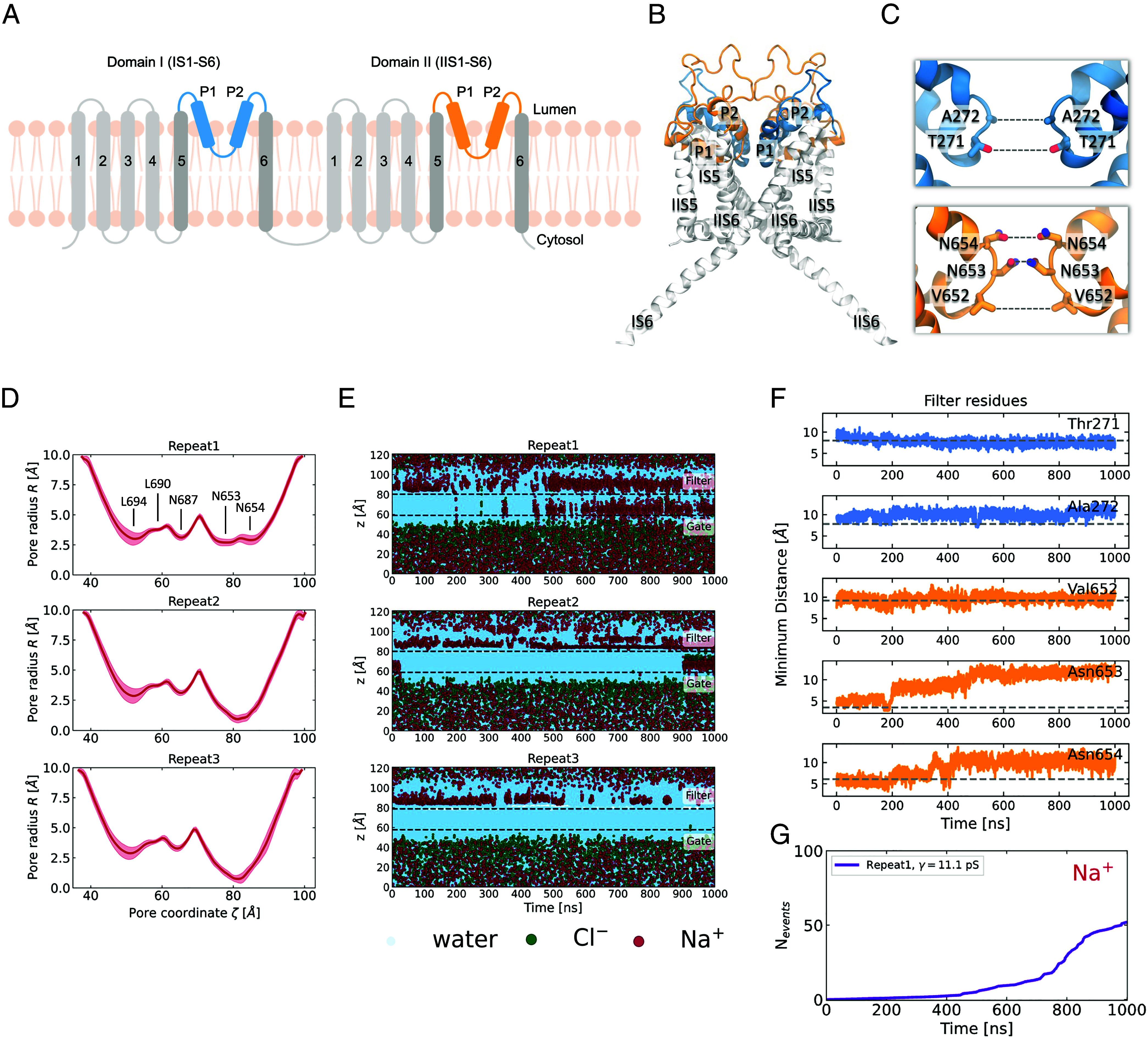
The selectivity filter undergoes a spontaneous conformational change and becomes more dilated. (*A*) Schematic representation of the architecture of human TPC2. The truncated protein that was used for the simulations did not include the transmembrane helices IS1-S4 and IIS1-S4, which are colored in light gray. The positionally restrained TM helices IS5-S6 are colored in dark gray and the unrestrained pore helices and selectivity filter are colored in blue (domain I) and orange (domain II). (*B*) Cartoon representation of the side view of the truncated TPC2 channel in the PI(3,5)P_2_-bound open state. The same color-coding is used as in the schematic representation. (*C*) Close-up of domain I (blue) and domain II (orange) of the SF. The SF-forming pairs of residues are labeled. (*D*) Mean pore radius profiles, computed for the last 300 ns of three independent trajectories with HOLE as implemented in the MDAnalysis package. The shaded regions indicate the SD. (*E*) Permeation events for water, Cl^−^, and Na^+^ as a function of time for three independent repeats at 750 mV potential. For the calculation, only the atoms that are found in a cylinder that encloses the ion conduction pore were taken into account. The cylinder is aligned with the direction of the channel pore, it is centered at the center of mass of the pore-forming residues and has a 6 Å radius. The center of mass of the selectivity filter and the cytosolic gate across the *z*-axis are indicated in dashed gray lines. (*F*) Time evolution of the minimum intersubunit separations of the five pairs of the SF-forming residues (as shown in *C*), for repeat 1. The dashed gray lines indicate the corresponding separations in the cryo-EM structure. There is clear increase in distance between the pairs of Asn653 and Asn654 residues. (*G*) Cumulative number of Na^+^ permeation events through the TPC2 channel, for repeat 1. The computed conductance value is shown in the legend.

Structurally, TPCs are homodimers where each protomer contains twelve membrane-spanning regions organized in two repeat domains (I and II) comprising a voltage-sensing region and pore (Fig. 1*A*) (9–12). They possess pseudo fourfold symmetry typical of the voltage-gated ion channel superfamily to which they belong and are likely evolutionary intermediates between one-domain and four domain members (13). Functionally, they were originally identified as Ca^2+^ channels gated by nicotinic acid adenine dinucleotide phosphate (NAADP) (14–16), a second messenger long known to release Ca^2+^ from the so-called “acidic Ca^2+^ stores” (17–19). Subsequent studies described TPCs as Na^+^ channels gated by the endo-lysosomally enriched signaling lipid, phosphatidylinositol 3,5-bisphosphate [PI(3,5)P_2_] (20). These apparently conflicting results were recently reconciled by the discovery that TPC2 has the highly unusual ability to switch its ion selectivity in ligand-dependent manner (21). NAADP renders TPC2 both Ca^2+^- and Na^+^-permeable whereas PI(3,5)P_2_ renders the channel largely Na^+^-selective. Coactivation of the channel with its ligands and newly described synthetic mimetics reveal further unusual activity whereby Ca^2+^ but not Na^+^ permeability is increased resulting in global Ca^2+^ signals through recruitment of IP_3_-sensitive channels on the endoplasmic reticulum (ER) (22, 23).

Single-channel recordings demonstrate gating of TPC2 by NAADP using a number of permeants including Ca^2+^ and Na^+^ (24, 25). But activation by NAADP is indirect through the NAADP binding proteins JPT2 and Lsm12 that associate with TPCs (26, 27), consistent with biophysically distinct inhibition of single TPC2 channels by the NAADP antagonist, Ned-19 relative to pore blockers (28). In contrast, PI(3,5)P_2_ binds directly to TPCs in domain I at the linker between the voltage sensor and pore as evidenced by cryo-EM structures of the mammalian isoforms, TPC1 (29) and TPC2 (30). Functional single-channel behavior of TPCs in response to PI(3,5)P_2_, however, is lacking reflecting a limited mechanistic understanding of ion permeation through TPCs, especially in the emerging context of polymodal gating and ion selectivity switching (31).

Here, we combined molecular dynamics simulations (MD) with electrophysiology to probe gating of TPC2 by PI(3,5)P_2_. We provide evidence that the selectivity filter (SF) of TPC2 is conformationally flexible and only supports ion flux when in a previously unobserved dilated state. The ion channel was more permeable to Na^+^ versus Ca^2+^, with a predicted Na^+^ conductance in agreement with direct measurements of single-channel activity. Our data establish a structural framework for understanding malleable ion permeation in TPCs and potentially other channels.

## Results

### The Cryo-EM Structure of TPC2 Does Not Allow Permeation of Ions.

We began by first trying to compute the conductance of human TPC2 using the PI(3,5)P_2_-bound open state structure (PDB: 6NQ0). A common practice in functional annotation studies by MD is to heavily restrain the backbone or Cα coordinates to prevent large deviations away from the experimentally derived model (32–36). We adopted this approach to keep the channel in the presumed open state and for expediency, we also truncated the protein to only keep the ion conduction pore domain (*Materials and Methods* and Fig. 1 *A*–*C*). We then performed MD simulations under transmembrane potentials ranging from 250 to 750 mV (luminal positive). To our surprise, we observed none to very few permeation events (*SI Appendix*, Fig. S1) even under the unrealistically large (750 mV) transmembrane potentials. The 6NQ0 structure was designated open based on the larger diameter at the activation gate (i.e., helix bundle crossing) relative to the designated closed PI(3,5)P_2_-bound state structure (PDB:6NQ2). However, our results suggest that it may be more representative of a “pre-open” state. To explore whether a possible increased flexibility of the SF could affect ion permeation, we set up simulations whereby the restraints were removed on the pore helices and the SF region (*Materials and Methods* and Fig. 1). However, once again, the number of permeation events was still extremely low except in one of the simulations (Fig. 1*E*). Closer inspection of this simulation revealed a dramatic change in the pore radius in the SF region (Fig. 1*D*) that corresponded to increased Na^+^ permeability. In repeat 1, for the first half of the simulation, the ions accumulated near the luminal side of the SF. However, after approximately 400 ns of simulation, ions began to frequently permeate through the SF into the pore and rapidly crossed the gate toward the cytosolic side. The timing of the increase in the number of permeation events was concomitant with the change in the SF radius (Fig. 1*G*). The change in the latter stems principally from an increase in separation between Asn653 and Asn654 pairs of residues from domain II which move away from each other in an asymmetric fashion (Fig. 1*F*). The first conformational change that occurs is that of the rotation of Asn653, which forms the narrowest constriction point across the channel, away from the central pore axis, followed by the upward rotation of one of the Asn654 residues towards the luminal side. Interestingly, the ions began permeating only when this change in Asn654 conformation was observed and they were able to engage and coordinate the Na^+^ ions (Movie S1). We term this newly observed conformation the Open-SF conformation. To investigate whether this conformational transition of the SF was a single random event, we performed an additional 12 repeats starting from the same cryo-EM state (analysis shown in *SI Appendix*, Figs. S2–S5). The SF displays a highly dynamic nature, and while in some simulations it remains in its initial state and blocks Na^+^ influx, in others it opens up to adopt the Open-SF conformation that was initially observed in repeat 1. We consistently observe that when this conformational transition occurs, the rotational motion of Asn653 and Asn654 leads to widening of the pore radius at the SF region and more Na^+^ ions start to permeate through the channel. In addition, we performed 16 × 100 ns replica exchange with solute tempering simulations [REST (37)—see *Materials and Methods*] to further investigate the dynamics of the filter. These simulations also revealed very similar conformations to the those found spontaneously in the conventional MD simulations (*SI Appendix*, Figs. S12 and S13), thus confirming that these alternative conformational states should be considered functionally relevant. Since the conformational transition of the SF can be attributed to the motion of Asn653 and Asn654, we chose the pair of the intersubunit distances of the γ-carbons of these two residues to inspect the probability density distribution of the filter being in the Closed-SF or the Open-SF conformation, analyzing the REST simulation at the lowest temperature (T = 310 K) (*SI Appendix*, Fig. S14). While the intersubunit Asn653 separation is *ca* 4 Å at the Closed-SF state, it spans an intermediate range between 6 and 9 Å, before the residues completely rotate outward and become separated by up to *ca* 12 Å. The Asn654 distance distribution is bimodal, with the two modes corresponding to the initial state where the two Asn654 face each other and the Open-SF state, when they rotate up toward the lumen.

### A Spontaneous Conformational Change in TPC2 Results in Na^+^ Permeation Events.

**Fig. 2.**
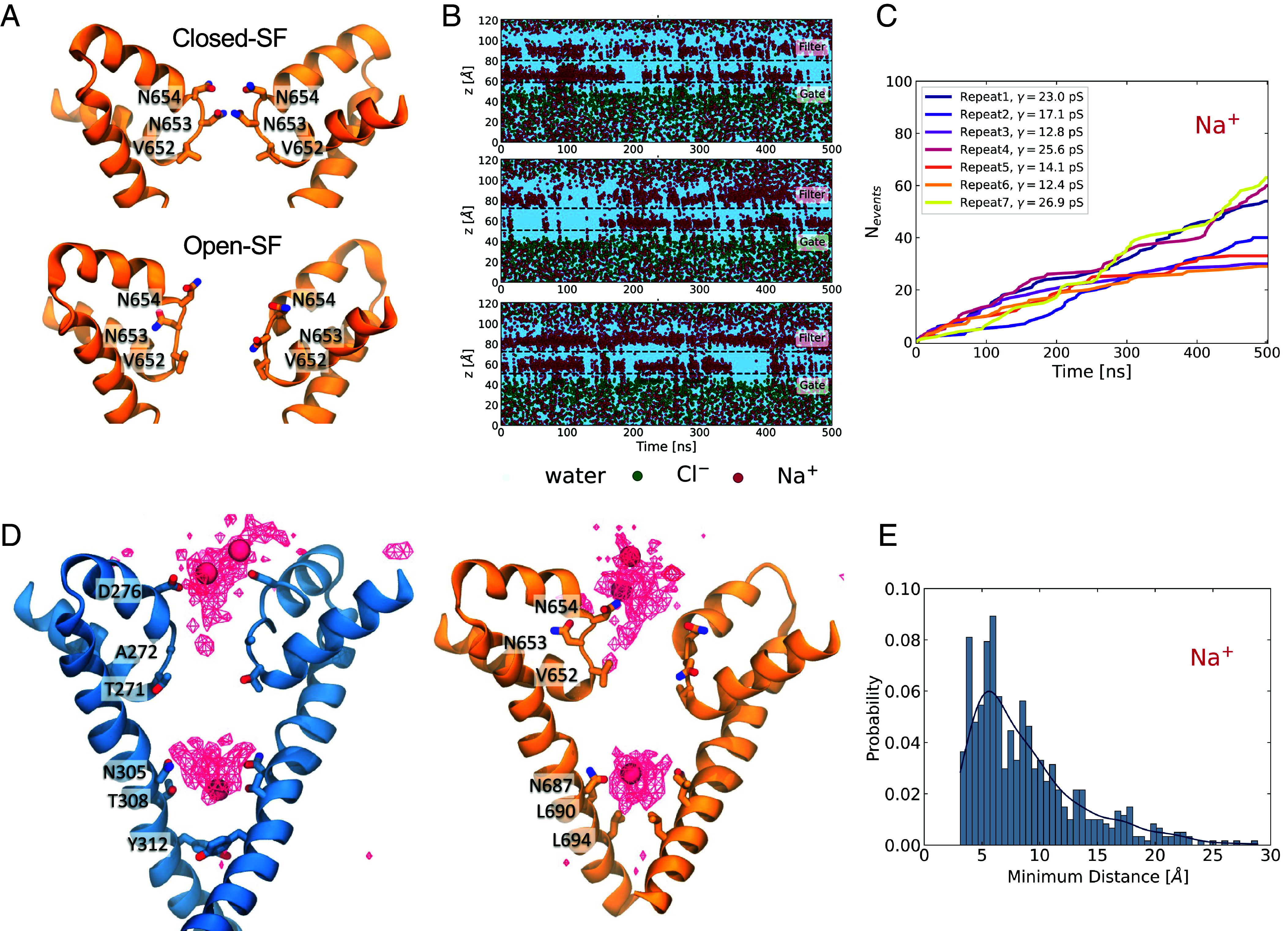
Nature of the conformational change within the SF. (*A*) Close-up of the domain II SF. The SF becomes more dilated, with the main conformational change observed at domain II. The first pair of asparagines (Asn654) rotate to point up toward the luminal side and the second pair of asparagines (Asn653) rotate outward in the opposite orientations. The shift in conformation is concomitant with the increase in permeation. (*B*) Three representative plots of the time evolution of the z-coordinates of the permeating water and ions. Starting from the open-SF conformation results in immediate and sustained permeation. (*C*) Cumulative number of Na^+^ permeation events for seven simulation repeats. (*D*) Na^+^ average density map indicates permeation pathway. Domains I (blue) and II (orange) are shown separately for clarity. The ion density and three permeating ions are shown as red wireframe and VdW representations, respectively. Two main binding sites are identified; the first is near the charged Asp276 and the polar Asn654, which point upward, engage and coordinate the motion of Na^+^. After the Na^+^ has crossed the SF into the channel, it is coordinated by two other pairs of asparagine residues, Asn308 and Asn687, where it remains engaged before crossing through to the cytosol. (*E*) Probability distribution of the distance between a Na^+^ bound at the SF and the nearest Na^+^ ion found at the luminal side of the channel.

Given this conformation resulted in a significant number of permeation events, we next instigated several more repeat simulations starting from this Open-SF conformation (Fig. 2*A*). In seven 500 ns-long simulations, the channel allowed a significant number of permeation events (Fig. 2 *B* and *C*, *SI Appendix*, Fig. S6, and Movie S2). The cumulative number of permeation events for each trajectory was used to compute the mean Na^+^ conductance (Fig. 2*C*). From these simulations, we predicted a conductance of 19 ± 6 pS. The pore radius profiles and selectivity conformations of all seven repeats are very similar (*SI Appendix*, Figs. S7 and S8).

Aggregation of the simulation data allowed us to visualize a “density” map of Na^+^ ions within the channel pore and to monitor the permeation pathway (Fig. 2*D*). The density map revealed two distinct locations where the ions appeared to spend a higher proportion of time during permeation. The first binding site was around and above Asp276 and Asn654, just above the SF. The slight asymmetry of the conformations of the two asparagines resulted in the ions mostly interacting with Asn654 of subunit A, which was more extended toward the luminal side. The permeating ions did not make contact with the side chains of Asn653 (*SI Appendix*, Fig. S9), which had rotated away from the pore. The second binding site was near two other pairs of asparagines, Asn305 (Domain I) and Asn687 (Domain II), which form part of the intracellular gate. The ions were coordinated by Asn305 and Asn687 before passing through the gate to the other side of the membrane. Notably, quite often both of these binding sites were occupied simultaneously, with one ion engaged at the SF region while the other was in the cavity, as shown in Fig. 2*D*. Previous studies on voltage-gated Na^+^ channels (32, 38) have explored the knock-on effect on the permeation mechanism, which occurs when a second ion is found in the vicinity of the first permeating ion and speeds up its permeation. In the case of TPC2, Fig. 2*E* shows that the most probable separation between a Na^+^ ion bound at the SF and its nearest Na^+^ in the region above the SF is approximately 6 Å, with an overall probability of this distance being lower than 7 Å of 48%. This result suggests that even though it is very likely that a second ion is present in close proximity to the permeating Na^+^ ion potentially accelerating its motion, permeation is still possible in the absence of nearby ions.

**Fig. 3.**
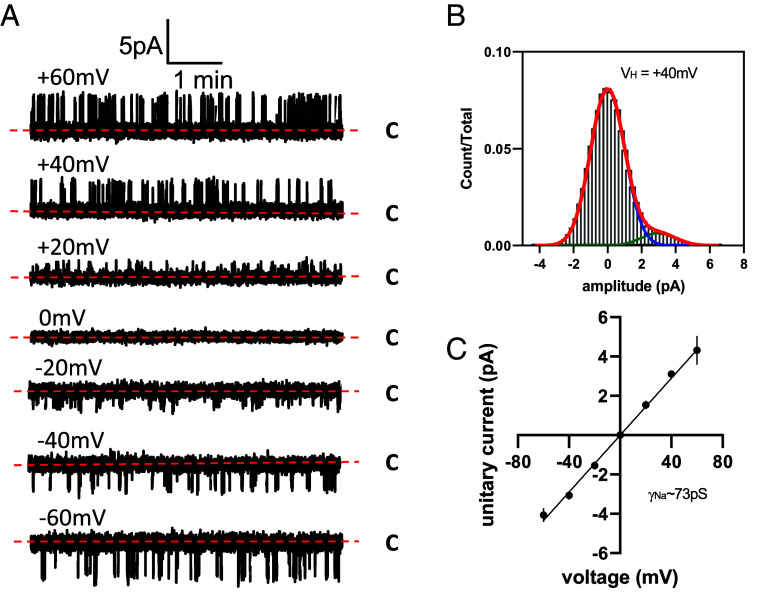
Unitary Na^+^ conductance of TPC2 activated by PI(3,5)P_2_. (*A*) Exemplar single-channel recordings of TPC2 rerouted to the plasma membrane of HEK-293 cells. C denotes closed current level and holding potentials are shown above corresponding traces. Currents were recorded in response to 10 μm of di-C8-PI(3,5)P_2_ added to the bath solution. (*B*) Typical current amplitude histogram for single TPC2-mediated currents at holding potential (V_H_) of +40 mV shown in panel *A*. (*C*) Current-voltage relationship for single TPC2 currents recorded at various holding potentials. Each data point indicates mean ± SEM (n = 3).

The unitary conductance of TPC2 stimulated with PI(3,5)P_2_ is not known. To address this experimental gap, we expressed TPC2 in HEK cells and rerouted it to the plasma membrane to facilitate patch-clamp analysis (Fig. 3). This was achieved by mutating the N-terminal targeting sequencing that normally directs it to the lysosome (25). Using symmetrical Na^+^ solutions, we resolved single-channel activity in excised patches in response to PI(3,5)P_2_ (Fig. 3 *A* and *B*). From analysis of current–voltage relationships, we determined a unitary conductance of 73 ± 3.8 pS (Fig. 3*C*), Thus, the predicted and experimentally derived conductances were in reasonable agreement.

### Free Energy of Na^+^ Permeation through the TPC2 Channel.

**Fig. 4.**
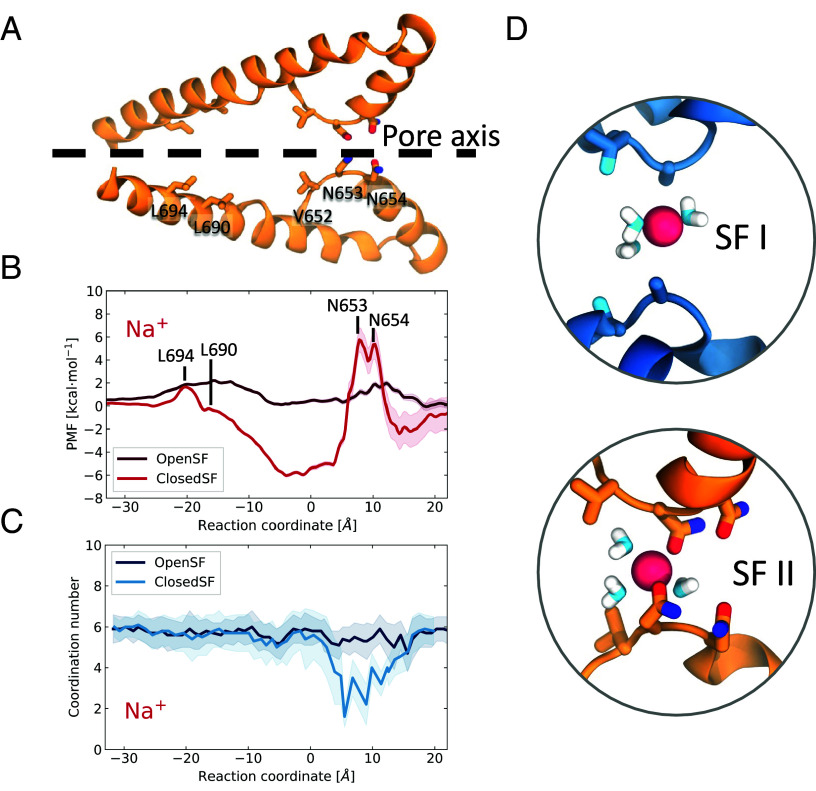
PMF profiles of Na^+^ permeation through TPC2. (*A*) Cartoon representation of the side view of domain II of the ion conduction pore. The side chains of the SF- and the gate-forming residues are shown in liquorice representations. (*B*) PMF profiles of Na^+^ permeation for the closed-SF cryo-EM structure (bright red) and the Open-SF structure (dark red), obtained from the MD simulations. The error was estimated with bootstrap analysis. The peaks in the energetic barriers are associated with the SF- and the gate-forming residues of domain II which are labeled. The cartoon representation in *A* has been aligned with the PMF to assist visualisation. (*C*) Na^+^ hydration during permeation as a function of the reaction coordinate. (*D*) Snapshots of a permeating Na^+^ when it crosses the Closed-SF; only three water molecules form its first hydration shell.

To investigate the influence of conformation on the energetics of permeation, we next conducted potential of mean force (PMF) calculations with Umbrella Sampling (Fig. 4*A*, *Materials and Methods*, and *SI Appendix*, Fig. S10). The PMF calculations for Na^+^ revealed that for the cryo-EM structure with the Closed-SF (Fig. 4*B*), there were significant barriers to permeation typically of the order of 8 kcal/mol, which are consistent with the low number of permeation events observed. The first energetic barrier that the ion faces during permeation is imposed by Asn654 and Asn653, as is expected given that these asparagines form two narrow constriction points. In the cavity between the SF and the gate, an energetic minimum suggests a favorable environment for Na^+^, which explains the long permeation time. Then, the ion has to escape an energy well of approximately 7 kcal/mol leading to the gate-forming residues Thr308 and Tyr312 (domain I) and Leu690 and Leu694 (domain II). For the Open-SF conformation, however, we observed a flattening of the PMF that results in barriers of approximately 2 kcal/mol, which explains the frequent permeation events.

Na^+^ permeation through the Closed-SF, although rare, results in partial dehydration of the ion, whose water coordination number drops to a minimum of 2 (Fig. 4 *C* and *D*), perhaps explaining why permeation events were so few under those conditions. In contrast, ion permeation through the Open-SF conformation leads to coordination numbers closer to 6 and never dips below 5 (and even then, only briefly). In both cases, the ions remain fully hydrated when passing through the activation gate at L690/L694.

### The Newly Observed TPC2 Conformation Is More Permeable to Na^+^ over Ca^2+^.

**Fig. 5.**
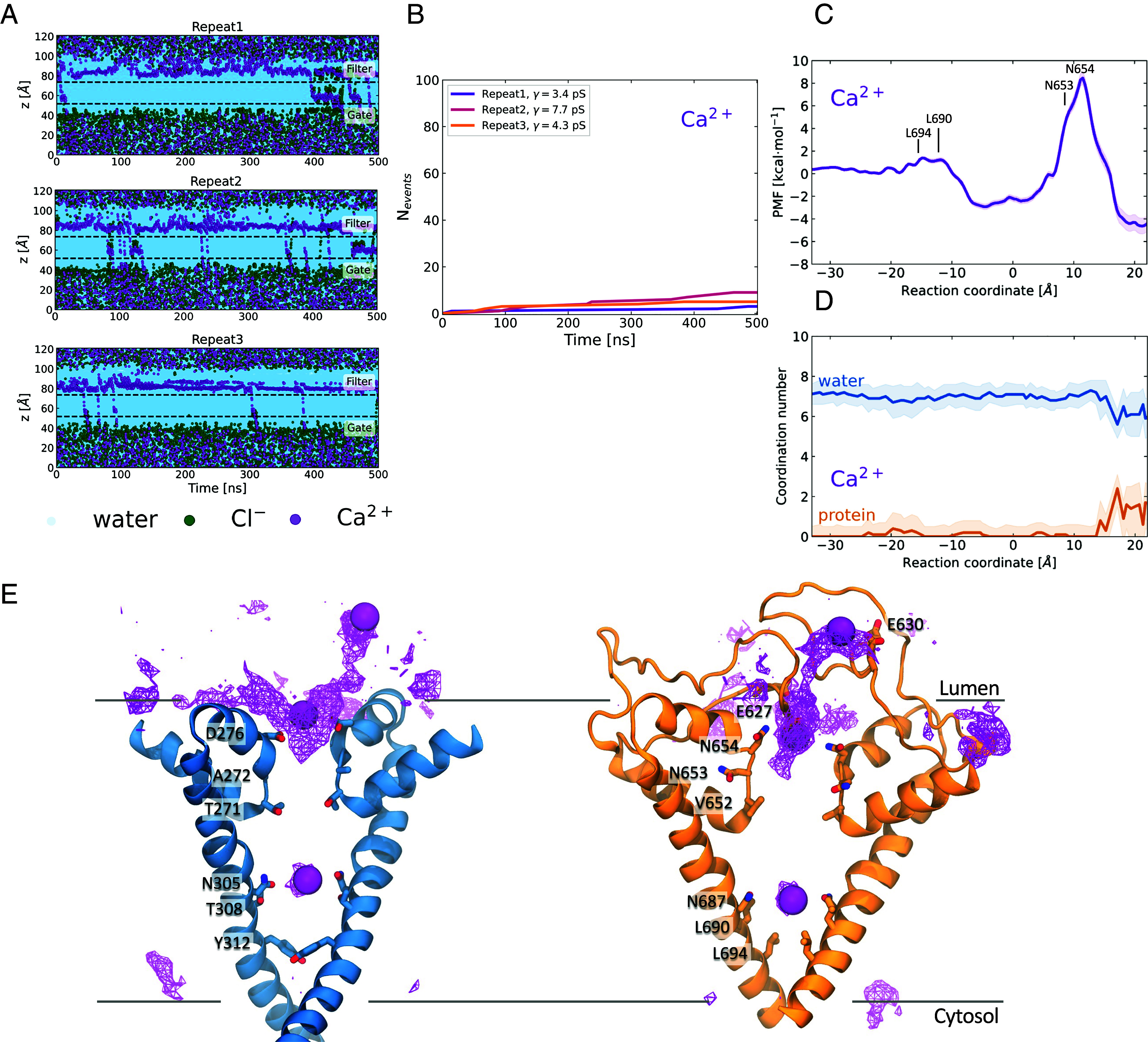
The PI(3,5)P_2_-bound open state of TPC2 is not permeable to Ca^2+^. (*A*) Permeation events for water, Cl^-^, and Ca^2+^ as a function of time for three independent repeats under 750 mV potential. For details on the calculation refer to Fig. 1. (*B*) Ca^2+^ cumulative number of permeation events, for all the simulation repeats. The computed ion conductance values are shown in the legend. (*C*) PMF for Ca^2+^ permeation through the TPC2 channel. (*D*) Ca^2+^ water (blue) and protein (orange) coordination numbers as a function of the reaction coordinate. Ca^2+^ is fully hydrated when it permeates the channel, except for the region before the SF, where it is in contact with the acidic residues Glu627 and Glu630. (*E*) Ca^2+^ average density map indicates permeation pathway. Domains I (blue) and II (orange) are shown separately for clarity. The ion density and three permeating ions are shown as purple wireframe and VdW representations, respectively. The Ca^2+^ ions accumulate in the region above the SF, where they are coordinated first by the Glu627 and Glu630 and then by Asp276 and Asn654.

The PI(3,5)P_2_-activated TPC2 is known to be Na^+^-selective and consequently was expected to be more permeable to Na^+^ than Ca^2+^ (21). We therefore set up simulations to investigate Ca^2+^ permeation. Starting from our Open-SF state conformation, we instigated three repeat simulations with Ca^2+^ as the permeant ion. We observed on average 5.7 Ca^2+^ transport events per 500 ns, resulting in a mean conductance of 5.1 ± 1.8 pS (Fig. 5 *A* and *B*). Considering that we recorded an average of 44.1 Na^+^ events per 500 ns, it is evident that Ca^2+^ permeability is significantly reduced in comparison to Na^+^, with a P_Na^+^_:P_Ca^2^^+^_ permeability ratio of 7.7:1. Indeed, contact analysis and the average density map indicated a different pattern of ion density for Ca^2+^ compared to Na^+^ (Fig. 5*E* and Movie S3). The highest ion density corresponded to the Ca^2+^ ions that aggregated above the SF, near the long IIS5-P1 linker. They were engaged by the acidic Glu630 and Glu627 and directed toward the SF where they are coordinated by Asp276, found in domain I, and by Asn654 in domain II. The ion density in the cavity between the SF and the activation gate can be attributed to the few Ca^2+^ ions that cross the SF and enter the channel, where they are coordinated by Asn305 (domain I) and Asn687 (domain II), similar to Na^+^.

The PMF profile of Ca^2+^ crossing through the TPC2 channel reveals a very high energetic barrier at the SF region of approximately 13 kcal/mol (Fig. 5*C*), indicating that this open-SF conformation, albeit permeable to Na^+^, is not permeable to Ca^2+^. Surprisingly, the ions were fully hydrated when passing through the SF with an average water coordination number of 7 (Fig. 5*D*); hence, the high barrier observed cannot be attributed to dehydration. The ions appeared partially dehydrated however, in the region above the SF, where they formed contacts with the carboxyl oxygen atoms of the acidic residues Glu627 and Asp276. These favorable coulombic interactions between Ca^2+^ and the acidic residues could explain the high energetic barrier observed. Indeed, a significant amount of energy would be required to disrupt these nonbonded interactions and lead to Ca^2+^ permeating through the SF and it would be worth further exploring the importance of Glu627 and Asp276 in the mechanism of Ca^2+^ permeation.

## Discussion

TPC2 channels exhibit agonist-dependent selectivity changes (39) that have been presumed to reflect changes in the conformation of the filter, suggesting that the SF has an inherent amount of dynamic flexibility. How this relates to permeability and selectivity is not well-understood. The recent cryo-EM structures of TPC2, including one structure (6NQ0) assigned as a possible open state, (30) provide a starting point to explore this further. We first assessed what predicted conductance could be obtained with the cryo-EM open-state structure. To our surprise, we could only observe a small number of permeation events when the filter was in a conformation close to the cryo-EM open state model, resulting in Na^+^ conductance values of only up to 5 pS.

In these simulations, we observed two modes of ion transport; in the first mode, a single ion crosses the SF and remains in the central cavity, coordinated by Asn305 and Asn687, for as long as 300 ns, whereas in the second mode, an ion remains coordinated by the same two pairs of asparagines in the central cavity, while a few others permeate through the pore. This route is reminiscent of the so-called “by-pass” mechanism (40) and has also been reported by Milenkovic et al., who performed MD simulations under transmembrane potential on the cryo-EM open-state PI(3,5)P_2_-bound TPC2 (41). They also recorded only a few ion transport events that induced similar conductance values (2 to 3 pS), in agreement with our initial observations, but not in line with the experimental conductance we report here. In our second attempt to explore ion transport, we removed the position restraints from the pore-helices and SF region, and as a result, the inherent conformational flexibility of the SF revealed itself in one of our simulations with a dramatic change in conformation. This Open-SF conformation almost instantaneously allowed the permeation of Na^+^ ions.

The permeation of ions in simulations initiated from the Open-SF conformation reproducibly resulted in Na^+^ conductance predictions that were in very good agreement with our experimental measurements. Little is known about the single-channel behavior of TPC2 in response to PI(3,5)P_2_. Such single-channel activity was reported recently (6), but only after stimulation with a specific synthetic TPC2 agonist (TPC2-A1-N). The nature of these currents is thus unclear and no current-voltage relationships were reported although unitary current size was similar to our measurements. The empirically derived unitary channel conductance for PI(3,5)P_2_ (~73 pS) reported here is similar to that for NAADP (~86 pS) (28). It is worth noting that MD simulations using conventional force fields tend to underestimate conductance rates (42, 43).

That Na^+^ is permeable in this Open-SF conformation, but not in the original cryo-EM conformation, is supported by the PMF calculations that indicate energy barriers of up to ~8 kcal/mol for the original cryo-EM conformation. This is also in agreement with barrier heights for Na^+^ in the Aradopsis thaliana Two Pore Channel 1 (AtTPC1) channel (44) (even though the filter residues are different). These barriers are considerably flattened in the new Open-SF conformation with maximum barriers closer to 2 kcal/mol, consistent with the conductance observed and indeed consistent with barrier heights previously observed for voltage-gated Na^+^ channels in recent works (45–47) as well as in older works [see Ing et al. for a review of some of the earlier studies (48)].

Nearly all permeation studies on sodium channels focused on those that possessed the canonical DEKA motif in the SF. These are clearly very different from the filter residues in TPC2, which is formed by two pairs of conserved asparagine residues. A key focus of these studies was the possibility and influence of ion knock-on, where the presence of a second Na^+^ ion in close proximity to the first Na^+^ ion has been postulated to influence the overall conduction rates. In our simulations under voltage, we computed a probability of ~0.5 for a second ion being in close proximity to the SF-bound ion before it crosses through to the central cavity. This observation along with the low energetic barriers of *ca* 2 kcal/mol imposed by the SF indicates that Na^+^ transport is possible without the presence of a second ion in the vicinity, but it could potentially be accelerated by it. We performed contact analysis between the permeating Na^+^ ion and the other Na^+^ ions in the umbrella sampling trajectories, for all the windows (*SI Appendix*, Fig. S11) and this showed that there are no Na^+^ ions in close proximity (<6 Å) that could potentially act in lowering the energy barrier of Na^+^ crossing. Thus, for TPC2 the mechanism of permeation differs from that observed in canonical Na^+^ channels.

Even though we observed significant permeation for Na^+^ ions, the conformational change is not particularly favorable for Ca^2+^ ions and we observe a relative permeability of around 7.7:1 in favor of Na^+^, whereas the experimental value is closer to P_Na_:P_Ca_ = 17:1 (49). The reason for this modest discrepancy could be that we are simply computing raw conductance in the absence of other ions. Experimentally, the bathing solutions are mixtures of ions. An outstanding question at this point is whether or not there are other open conformations of the SF and in particular whether these are more permeable to Ca^2+^. Structural studies of TPC2 bound with NAADP along with one of its binding proteins, JPT2 or LSM12 (26) or of TPC2 bound with the synthetic agonist TPC2-A1-N, will most likely be needed to resolve that.

During the preparation of this manuscript, a preprint was published (50) that used a similar simulation strategy to examine ion permeation properties of TPC2. The authors observed a conformational transition of the SF reminiscent of that reported here that was permeable to Na^+^ but less so to Ca^2+^ (P_Na_:P_Ca_ = 9.4:1). Consistent with our observations, Ca^2+^ ions had higher resistance times near charged residues on the luminal surface that likely contributed to the selective passage of Na^+^ ions.

The idea of functional plasticity likely extends beyond TPC channels to the Transient Receptor Potential (TRP) channel family (51). For example, the TRPV1 channel shows changes in Ca^2+^ permeability that have been attributed to changes in the SF (52)—a suggestion later supported by cryo-EM structures (51). Our understanding of the factors that control selectivity in ion channels is constantly being refined [for an excellent review see Flood et al. (40)]. Conformational dynamics of the filter is certainly one key aspect that defines selectivity, but in vivo, the system is far from equilibrium and many different ion types are present. It will certainly be of interest to explore what role, if any, other ions have on the conductive state of TPC channels, as has been discussed for other Na^+^ channels (53).

In summary, we have revealed that the SF of TPC2 shows considerable conformational dynamics and that this appears necessary in order that the channel can adopt an asymmetric conformation for the preferential permeation of Na^+^ ions. Why this state has not been captured in the cryo-EM remains unclear, but one possibility is that only subtle changes in environment are needed to change the distribution of conformational states and the structure observed is simply one of many accessible states. Indeed, whether there are other distinct conformations that favor Ca^2+^ permeation also remains to be discovered. Regardless, it is clear that TPC2 channels can achieve selectivity via a very different mechanism compared to other Na^+^ channels (54).

## Materials and Methods

### MD Simulations.

The PI(3,5)P_2_-bound open state of human TPC2, solved with cryo-EM at a resolution of 3.7 Å (PDB ID: 6NQ0) (30), was used as the starting point for the MD simulations. The ligands were removed, and the protein structure was truncated to retain only the ion conduction pore [residues 200 to 346 (domain I) and residues 562 to 701 (domain II)]. The system was prepared using the CHARMM-GUI Membrane Builder (55, 56). The pKa values were calculated using PROPKA3.0 (57), which predicted standard protonation states of the protein residues. Careful examination of the solvent-exposed titratable residues located at the luminal side of the protein revealed that these residues are predicted to retain standard protonation states even in the lower-pH environment of the lumen. The protein was embedded in a 1-palmitoyl-2-oleoyl-sn-glycero-3-phosphocholine1-palmitoyl-2-oleoyl-sn-glycero-3-phosphocholine (POPC) bilayer and solvated and either NaCl or CaCl_2_ were added to neutralize the system and induce the physiological ion concentration of 0.15 M. The CHARMM36m force field (58) was used for the protein and the TIP3P model (59) was employed for the water. The chloride and sodium ions were represented with the CHARMM model, whereas the calcium ions were represented with the multisite model developed by Zhang et al. The CHARMM-GUI protocol (55, 56) was adopted for system equilibration. For the production simulations, a transmembrane potential of either 500 or 750 mV was applied with direction from the luminal side to the cytosolic side. Periodic boundary conditions were applied in all three dimensions. The hydrogen-containing bonds were constrained using the LINCS algorithm. The time-step was 2 fs and the leap-frog algorithm was selected for integration of the equations of motion. Coordinates were saved every 100 ps. The temperature was maintained at 310 K with the v-rescale thermostat (60) and a coupling constant of 0.5 ps, whereas the target pressure was maintained at 1 bar with the Parrinello–Rahman barostat (61) and a coupling constant of 5 ps. The particle mesh Ewald summation (62, 63) was adopted for treatment of reciprocal-space electrostatic interactions with a cubic interpolation and a grid spacing of 0.12 nm. The van der Waals interactions were smoothly switched off from 1.0 nm to 1.2 nm. In the first set of simulations, all the α-carbon atoms of the protein were position-restrained by applying a soft harmonic potential with a force constant of 1,000 kJ mol^−1^ nm^−2^, to ensure that the channel remains in the experimentally resolved open-state. In the second set of simulations, the harmonic restraints were removed from the pore helices and the SF [residues 240 to 287 (domain I) and 600 to 669 (domain II)], to allow for conformational flexibility upon ion permeation. For each system, 2 to 15 repeats of 500-ns- or 1,000-ns- long simulations were performed, amounting to a total simulation time of 18 µs.

### Umbrella Sampling Simulations.

PMF calculations were performed to obtain the free energy of ion permeation through the TPC2 channel pore. The PMF profiles were obtained using the umbrella sampling method where either a sodium or a calcium ion were kept harmonically restrained across the pore axis in windows of a 1-Å spacing. The force constant was 2,500 kJ mol^−1^nm^−2^ near the SF region where the energetic barrier was higher and 1,000 kJ mol^−1^nm^−2^ everywhere else. The reaction coordinate ξ was defined as the ion distance along the z-axis from the protein center of mass, with values ranging from −35 to +22 Å. The starting coordinates were generated by swapping a water molecule positioned on the axis that passes through the center of the pore and at ξ = +22 Å and then gradually moving it along the z-axis with 1-Å steps. A minimization step was performed for each system with the permeating ion fixed, to allow water to equilibrate around it. For the production runs, the same parameters that were described in the previous section were adopted. The PMF simulations were carried out for 20 ns each and during analysis, the first 2 ns were disregarded as equilibration time. The PMF profiles were obtained with the weighted histogram analysis method (64) and 100 bootstraps were used for estimation of statistical uncertainty. The total simulation time amounted to approximately 4 μs.

### REST Simulations.

Replica exchange with solute tempering is a variant of the replica exchange method, but tempering is only applied in a selected region of interest, to allow for adequate conformational sampling with fewer replicas (37). In our REST simulations, the SF and the pore-helix region [residues 240 to 287 (domain I) and 600 to 669 (domain II)] were selected to apply tempering, whereas the rest of the protein was kept in the initial temperature of 310 K. A total of 16 REST simulations were performed, spanning an effective temperature of 310 to 1,000 K. Replica exchange was attempted every 1,000 steps. The simulation length is 100 ns per replica. The REST analysis is included in *SI Appendix*.

MD simulations were performed with the GROMACS software package (65), version 2021 and the REST simulations were conducted using GROMACS, version 2021, patched with PLUMED, version 2.7 (66).

### Data Analysis.

The pore radius profiles of the channels were calculated using the HOLE program (67), implemented in the MDAnalysis package. The channel conductance of permeating ions with charge *Q_ion_* was calculated based on the number of permeation events (*N_event_*) over the entire trajectory. A permeation event was defined as one ion permeating through the cytosolic gate of the channel from the luminal side to the cytosolic side. The conductance (*C*) of a trajectory of a certain time length (*t*) under transmembrane potential (*V_tm_*) was calculated as follows:

$$C=\frac{I}{V_{\mathrm{tm}}}=\frac{N_{\mathrm{event}}\times Q_{\mathrm{ion}}}{t\times V_{\mathrm{tm}}}.$$

Coordination numbers were defined as the number of water oxygen atoms within 4.5 Å of Na^+^ or Ca^2+^, the positions of the first minimum in the bulk ion-water oxygen radial distribution function.

In-built GROMACS tools, in-house Python scripts, and the MDAnalysis package (68, 69) were used for trajectory analysis. Visual Molecular Dynamics (VMD) (70) was used for visualization and image preparation.

### Single-Channel Recordings.

Currents were recorded in the inside-out configuration from patches excised from the plasma membrane of HEK-293T stably expressing stably expressing human TPC2^L11/L12A^-monomeric red fluorescent protein (mRFP) (71) using symmetrical Na^+^ as charge carrier as done previously (31). The pipette (luminal) solution contained (in mM):145 NaCl, 5 KCl, 1 MgCl_2_, 2 CaCl_2_, 10 Hepes, and 10 2-(N-morpholino)ethanesulfonic acid (MES) (pH adjusted to 4.6 using methane sulfonic acid). The bath (cytosolic) solution contained (in mM): 145 NaCl, 5 KCl, and 10 4-(2-hydroxyethyl)-1-piperazineethanesulfonic acid (HEPES) (pH adjusted to 7.2 using NaOH). PI(3,5)P_2_ (10 μm, diC8 form) was from Echelon Biosciences and added to the bath solution via a pressurized perfusion system (AutoMate Scientific). All electrophysiological recordings were made at 23 °C. Acquisition and analyses of single-channel recordings were done as described previously (28).

## Supplementary Material

Appendix 01 (PDF)

Movie S1.

Movie S2.

Movie S3.

## Data Availability

MD simulation data have been deposited in Zenodo (DOI:10.5281/zenodo.12742282) (72). All other data are included in the article and/or supporting information.
