# Supplementary material for: Plasticity of the selectivity filter is essential for permeation in lysosomal TPC2 channels
Source: Proc Natl Acad Sci U S A. 2024 Jul 29;121(32):e2320153121. doi: 10.1073/pnas.2320153121 (PMC11317647; doi:10.1073/pnas.2320153121)
Supplement: Supplementary file 1 — Appendix 01 (PDF) [file pnas.2320153121.sapp.pdf]

SUPPORTING INFORMATION

Plasticity of the selectivity filter is essential for permeation in lysosomal TPC2 channels.

Afroditi-Maria Zaki[1], Süleyman Selim Çinaroġlu[1†], Taufiq Rahman[2], Sandip Patel[3] and Philip C. Biggin[1].*

[1] Structural Bioinformatics and Computational Biochemistry, Department of Biochemistry, University of Oxford, Oxford, OX1 3QU, UK

[2]Department of Pharmacology, University of Cambridge, Cambridge CB2 1PD, United Kingdom

[3]Department of Cell and Developmental Biology, UCL, London WC1E , 6BT, UK

[†]Present address: Department of Bioengineering, Ege University, Bornova, 35040, Türkiye

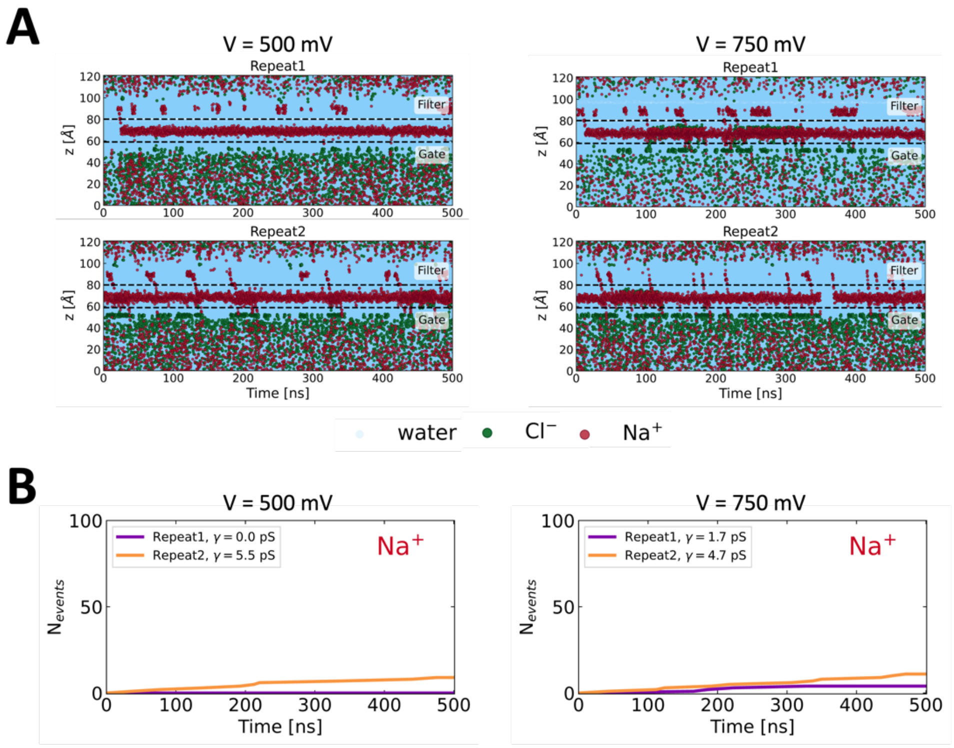

***Figure 1S**. (**A**) Permeation events for water, Cl^- and Na^+ as a function of time at 500 mV (left) or 750 mV potential (right), for the Cryo-EM PI(3,5)P_2-bound open state of TPC2. The center of mass of the selectivity filter and the cytosolic gate across the z-axis are indicated in dashed grey lines. For details on the calculation, refer to Fig. 1. At 500 mV, in Repeat 1, one ion permeates through the SF and remains in the channel cavity for the rest of the simulation time, more than 450 ns. In Repeat 2, one ion remains bound at the channel cavity, while a few other ions are simultaneously crossing the channel. (**B**) Cumulative permeation events for Na^+ permeation at 500 mV (left) and 750 mV (right). The computed conductance values for each simulation repeat are shown in the legends. Either zero or a few permeation events are recorded, resulting in very low conductance of up to 5.5 pS.*

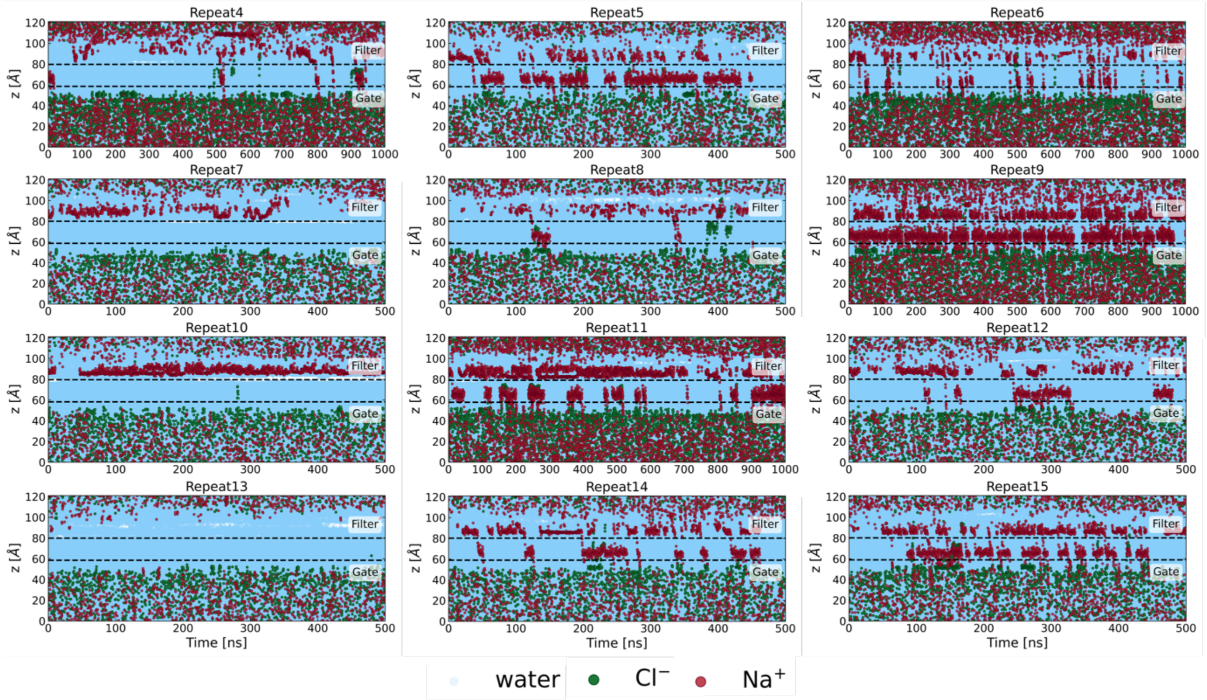

Figure 2S. *Permeation events for water, Cl^- and Na^+ as a function of time at 750 mV potential, for the Cryo-EM PI(3,5)P_2-bound open state of TPC2, where the selectivity filter and the pore helices are unrestrained, while the rest of the protein's Cα atoms are position-restrained. The center of mass of the selectivity filter and the cytosolic gate across the z-axis are indicated in dashed grey lines. For details on the calculation, refer to Fig. 1. In the main manuscript, three representative repeats of this set of simulations are presented, but 15 more were performed and included here. The data reveal a range of permeation patterns for sodium, where in some repeats it does not cross the ion channel, whereas in other (e.g. see Repeat 5 or Repeat 9) frequent permeation events are monitored.*

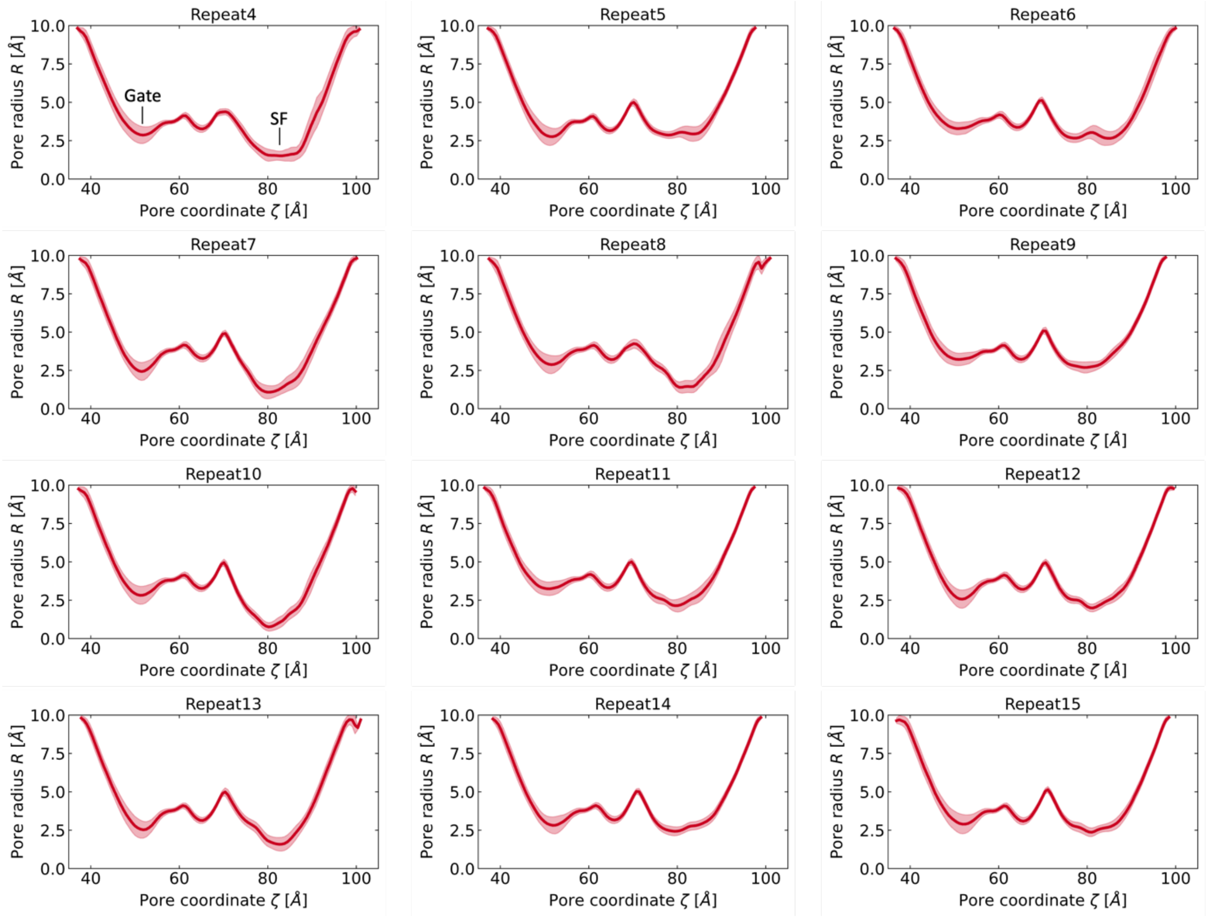

Figure 3S. *Mean pore radius profiles, computed for the last 300 ns of the 12 simulations described in Fig. 2S. The shaded regions indicate the standard deviation. The pore radius at the SF region (labelled in the Repeat4) exhibits values ranging between 1-3 Å approximately. The increase in the SF region radius is directly correlated with the increase of sodium permeation (see Fig. 1 and 2S).*

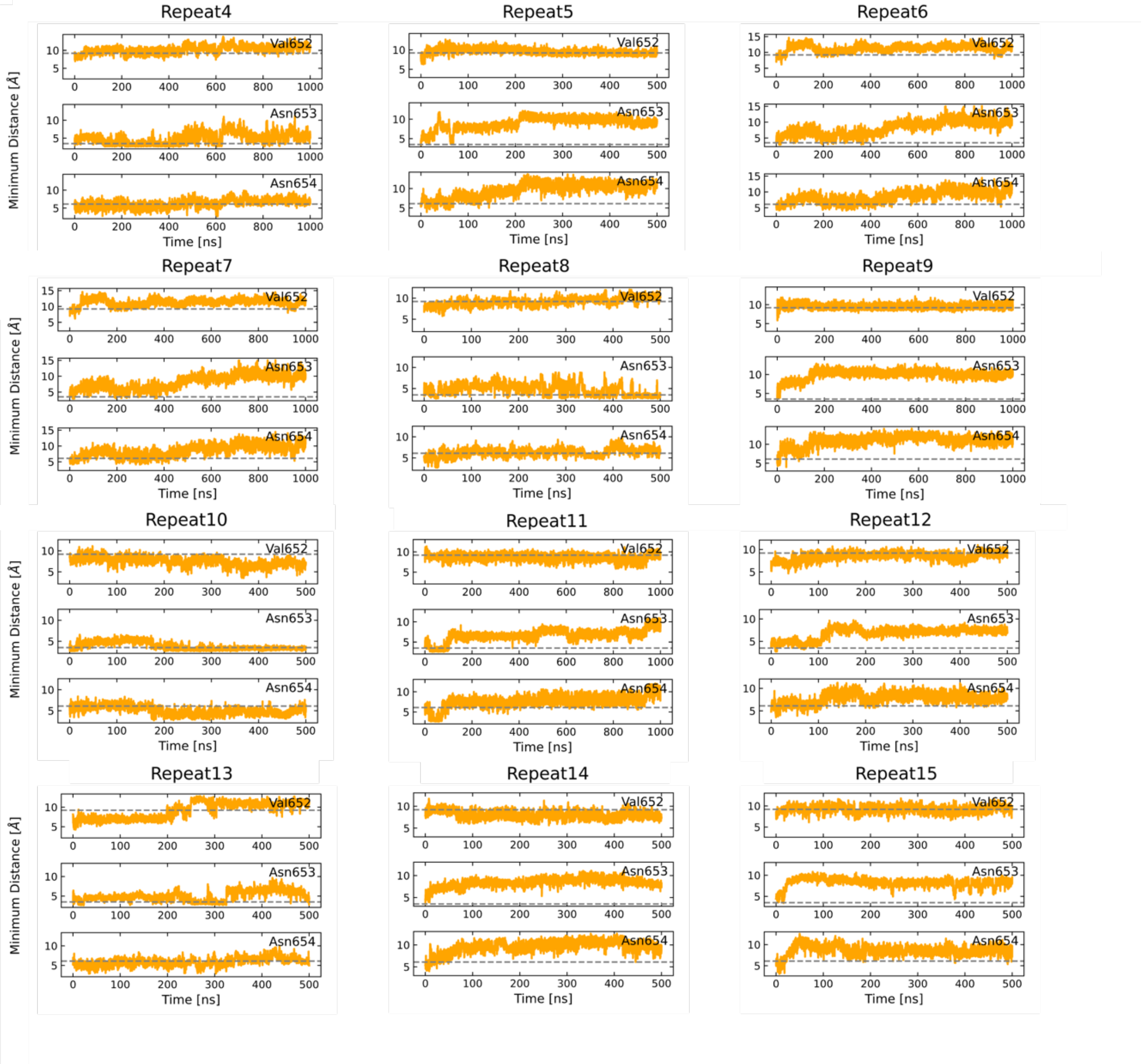

Figure 4S. *Intersubunit minimum distances for the SF-forming residues of domain II, for the simulations described in Fig 2S. In some of the repeats (e.g. see repeat5 or repeat11) the intersubunit separation increases as Asn653 and Asn654 move apart and the SF transitions to a more dilated conformation. This change in the conformation of these two key asparagines is a prerequisite for the increase in sodium permeation (see Fig. 1 and Fig. 2S).*

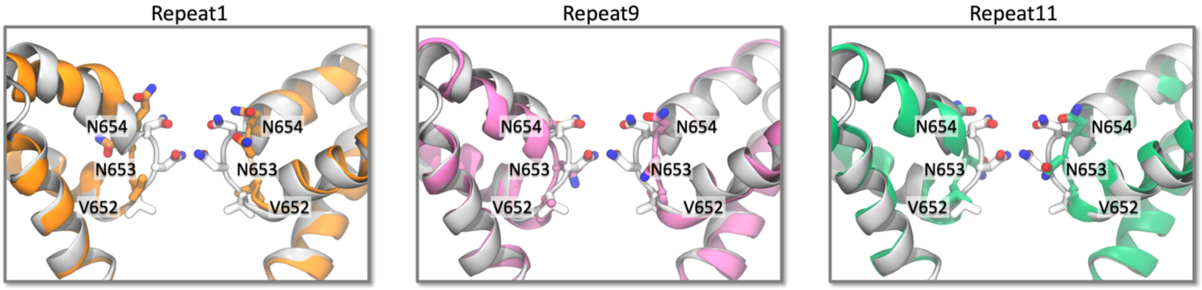

Figure 5S. *Snapshots of the domain II SF region adopting a dilated conformation ('Open-SF') as seen in three simulation repeats (Repeat 1: orange, Repeat 9: mauve and Repeat 11: green), at 750 mV, as explained in Fig. 1 and 2S. The dilated SF are overlaid with the starting cryo-EM PI(3,5)P_2-bound SF structure, shown in white and labelled in the manuscript as 'Closed-SF'. In our simulations, we can repeatedly see the SF revisiting a dilated conformation with Asn654 rotating upwards to the luminal side and Asn653 rotating outwards, away from the pore center. This conformational change is necessary to allow sodium permeation.*

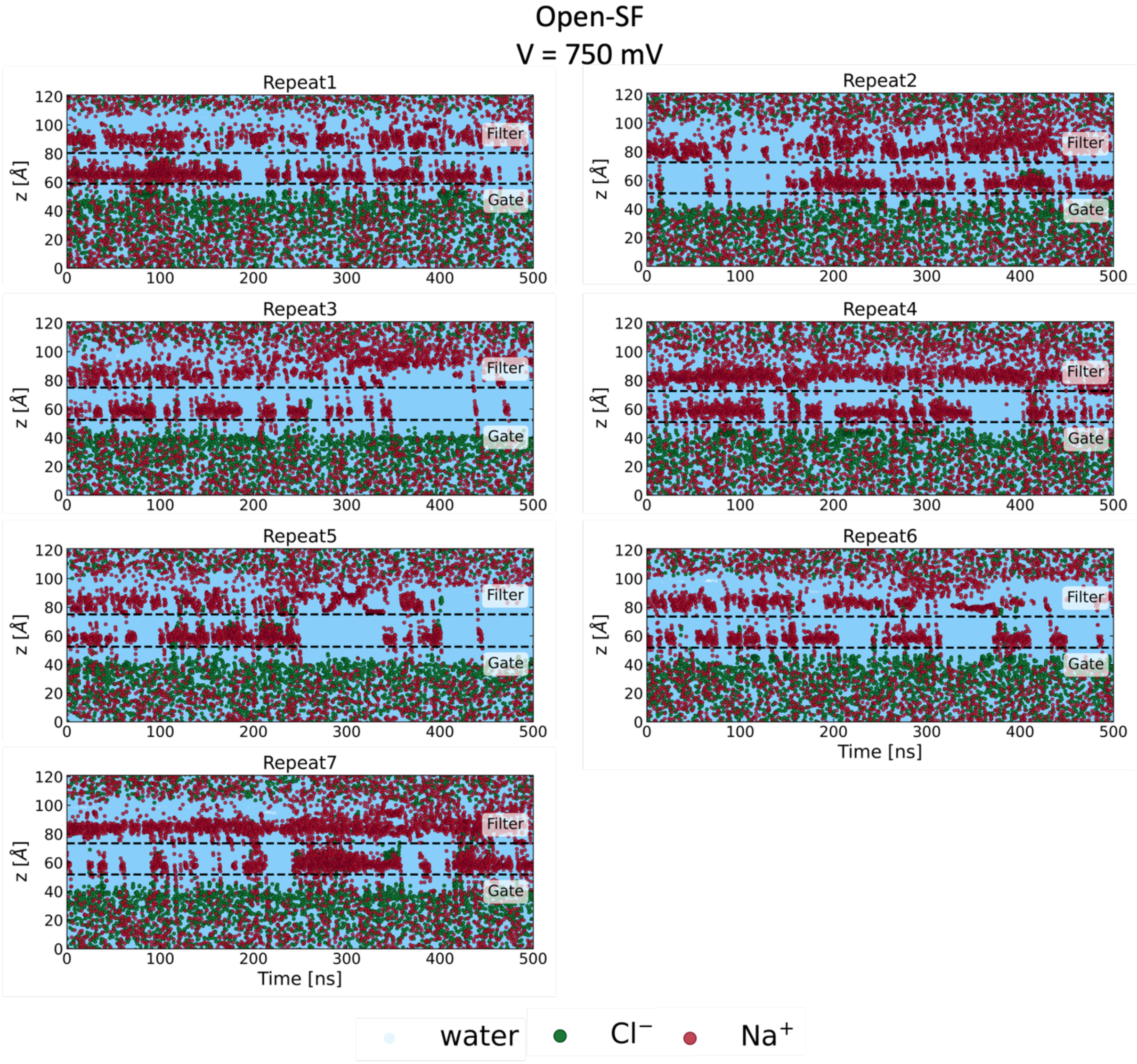

Figure 6S*. Permeation events for water, Cl⁻ and Na⁺ as a function of time at 750 mV potential, for the newly obtained Open-SF conformation of TPC2. The center of mass of the selectivity filter and the*

cytosolic gate across the z-axis are indicated in dashed grey lines. Data are obtained for seven 500ns-long simulation repeats. Frequent Na^+ transport events are recorded, for all repeats, suggesting that the SF remains dilated and renders the TPC2 channel Na^+-conductive.

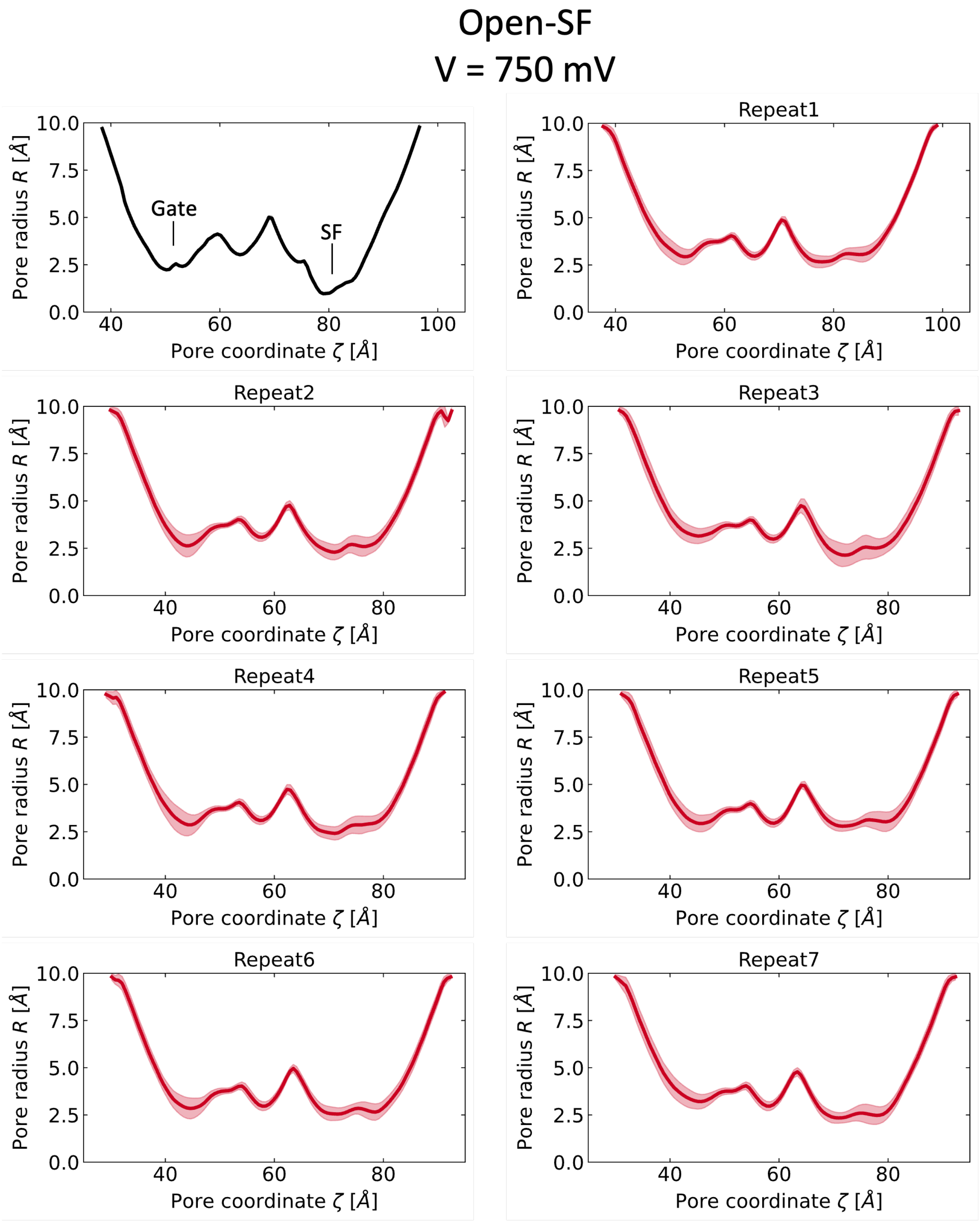

***Figure 7S**. Mean pore radius profiles, computed for the last 300 ns of the simulations of the newly obtained Open-SF conformation of TPC2. The shaded regions indicate the standard deviation. The top left plot shows the pore radius profile of the starting cryo-EM PI(3,5)P_2-bound open state, for comparison and the gate and SF regions are labelled. The pore at the SF region has a radius of approximately 2.5 Å in all simulations, increased in comparison to the 1-Å radius of the starting cryo-EM structure, suggesting that the dilated-SF state is stable throughout the trajectories.*

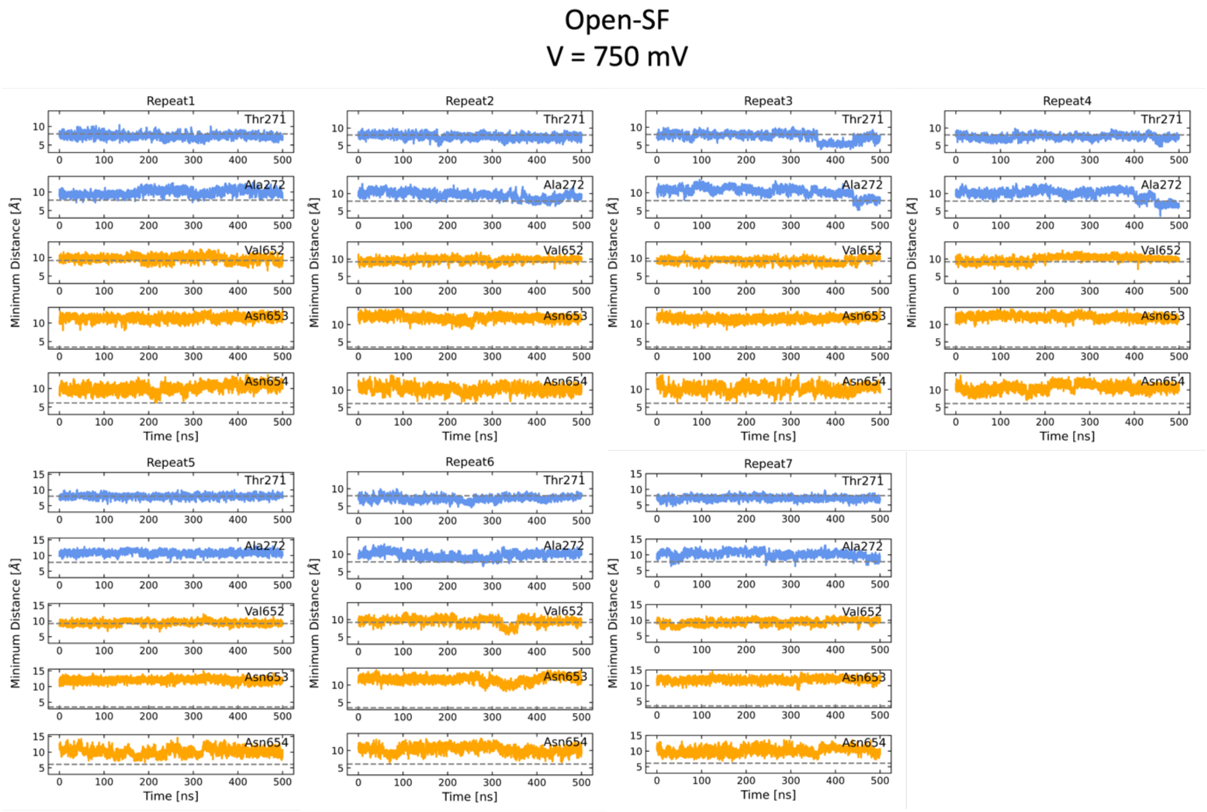

Figure 8S. *Time evolution of the intersubunit minimum distances of the SF-forming residues of domain I (blue) and domain II (orange) for the simulations starting from the Open-SF conformation of TPC2. The distances between Asn653 and Asn654 remain increased in comparison to the corresponding distances of the cryo-EM PI(3,5)P_2-bound state (grey dashed lines), suggesting stability of the dilated-SF state.*

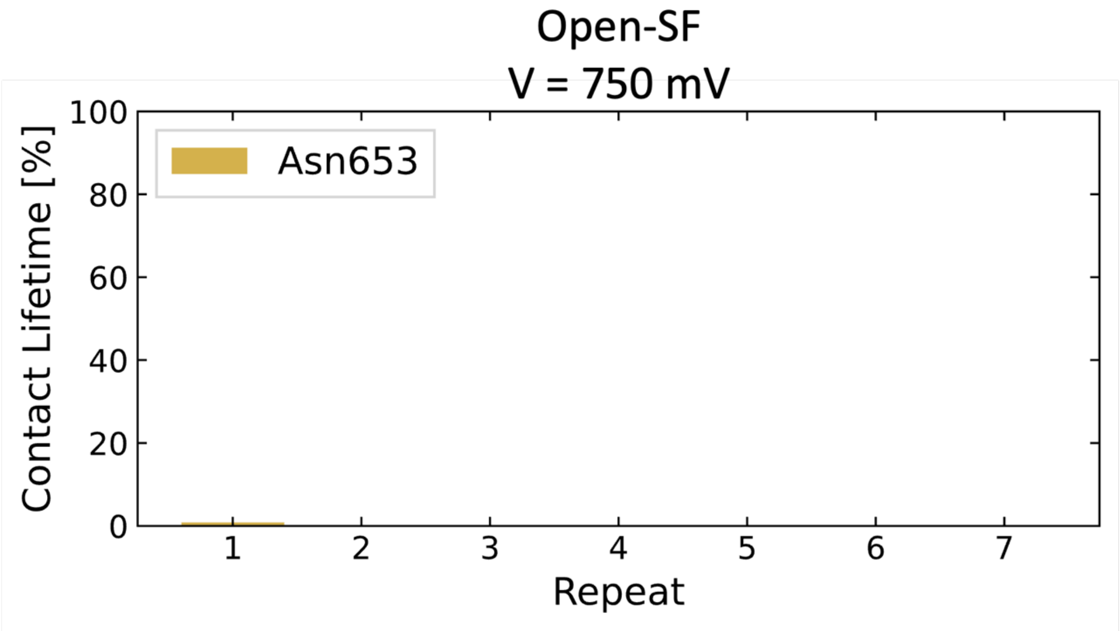

Figure 9S. *Contact lifetime computed between the side chain of the key SF residue Asn653 and all the sodium ions, for the simulations starting from the Open-SF conformation. A contact was recorded when the separation between Asn653 and a sodium ion was within 6 Å. The contact lifetime was computed as the ratio between the number of frames where a contact was present over the total number of frames. Almost no contacts at all are observed and this is due to the position of Asn653 side-chain, which has rotated outwards, away from the pore center.*

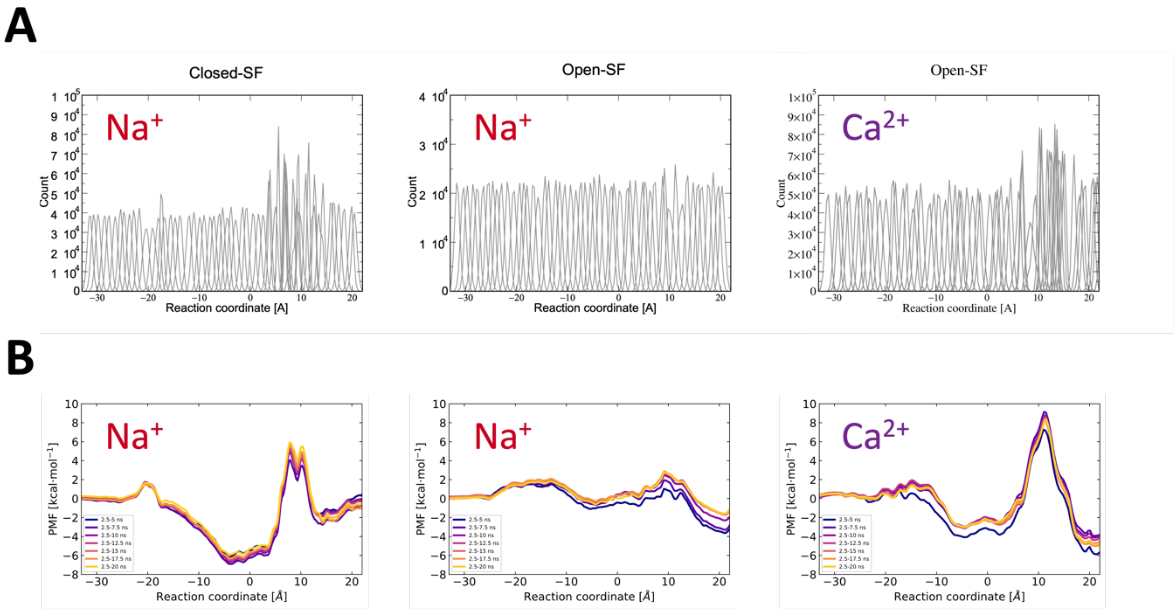

***Figure 10S**. (**A**) Umbrella sampling histograms for the Na^+ and Ca^{2+} ion permeation potential of mean force profiles, obtained for the cryo-EM Closed-SF TPC2 structure (left) and for the Open-SF TPC2 structure obtained from the MD simulations (center, Na^+ and right, Ca^{2+}). For details on the PMF calculation refer to **Methods**. The sufficient overlap between histograms of adjacent windows allows the accurate calculation of the free energy of ion transport through the channel. (**B**) Convergence of the PMF profiles with respect to simulation time. The first 2.5 ns were discarded as equilibration time. All PMF profiles have converged after 10 ns.*

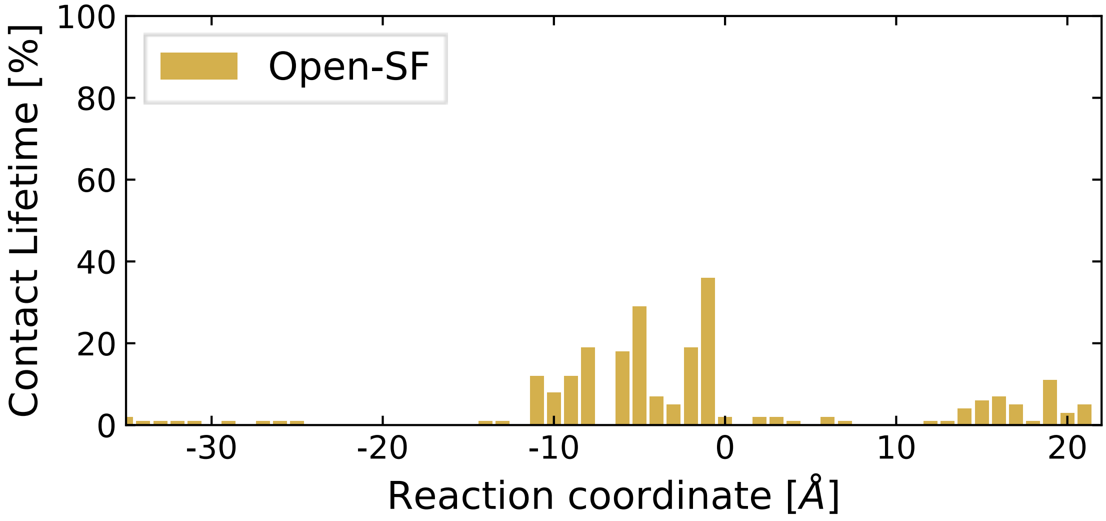

Figure 11S. *Sodium-sodium contact lifetime computed between the permeating sodium ion and all other sodium ions in the system, for the PMF of the Open-SF case. A contact was recorded when the separation between the permeating ion and another sodium ion was within 6 Å. The contact lifetime was computed as the ratio between the number of frames were a sodium-sodium contact was present over the total number of frames. The calculation was repeated for all the umbrella sampling trajectories across the reaction coordinate, ξ. For ξ ranging between 5-15 Å (the region that corresponds to the SF (see Fig. 4B)), almost no contacts are formed. This is evidence that the conformational change of the SF alone results in flattening of the free energy barrier without the requirement that other sodium ions are found in close proximity. The region where ξ is ranging between -11-0 Å corresponds to the channel cavity where the permeating ion can be in proximity with a second sodium ion that is entering the channel at the SF.*

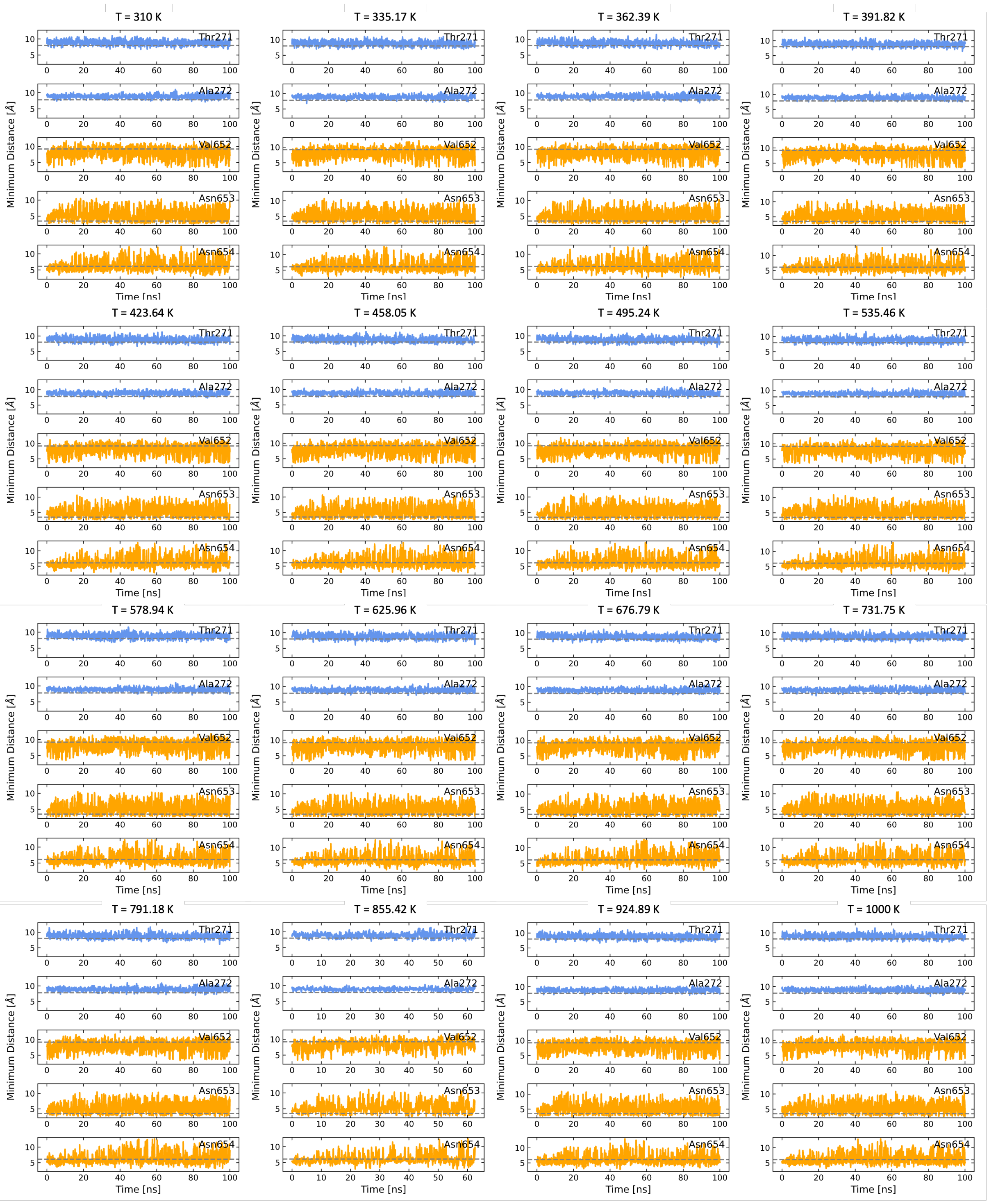

Figure 12S. *Time evolution of the intersubunit minimum distances of the SF-forming residues of Domain I (blue) and Domain II (orange) for the 16 replicas of the REST simulations. The distances between Val652, Asn653 and Asn654 fluctuate rapidly and deviate from the initial distances, which correspond to the cryo-EM PI(3,5)P_2-bound open state (grey dashed lines) and the SF frequently transitions between the Closed-SF and Open-SF conformation.*

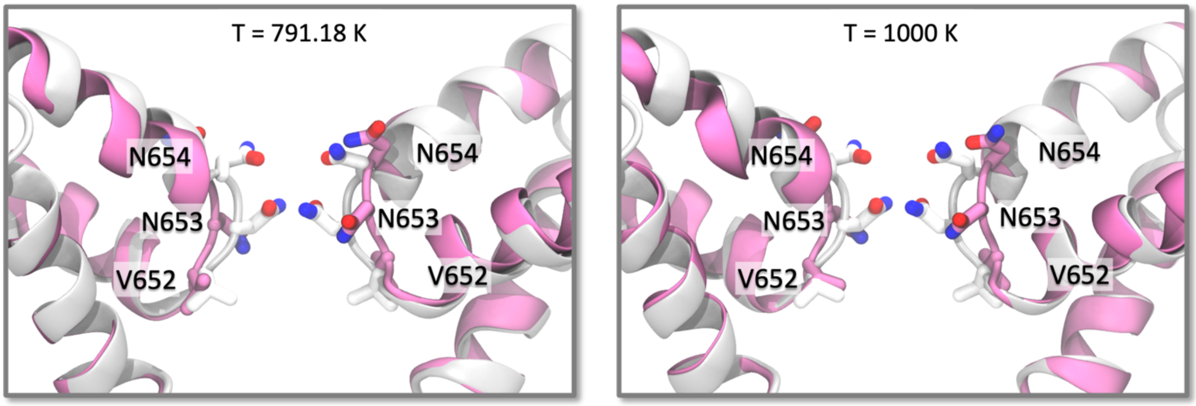

Figure 13S. *Representative snapshots of the dilated state of the SF, observed in the REST trajectories in T=791.18 K (left) and T=1000 K (right) shown in mauve, overlaid with the starting cryo-EM PI(3,5)P_2-bound state in white. The key SF-forming domain II residues are shown in licorice and are labeled. The SF has undergone a conformational change, similar to the one observed with the MD simulations under transmembrane potential, where the two Asn654 have rotated upwards and the two Asn653 move away from the center of the channel, widening the pore.*

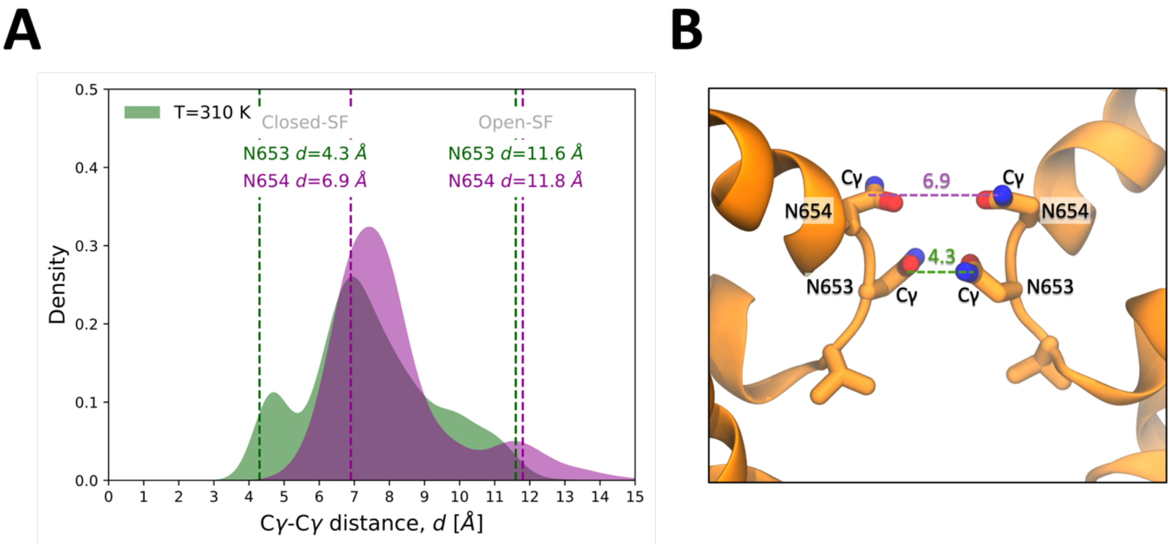

Figure 14S. (A) *Probability density distribution of the inter-subunit N653-N653 (green) and N654-N654 (magenta) distances, measured from their respective γ-carbons, for the REST simulation at T=310 K. The reference distances for the Closed-SF structure (PDB: 6NQ0) and the Open-SF structure (representative snapshot taken from the MD simulations under transmembrane voltage) are labelled and shown in dashed lines, for comparison.* ***(B)*** *The reference Closed-SF structure, showing the Cγ-Cγ separation between N653 and N654.*
